# Multilocus Sequence Typing Analysis of Invasive and Non-Invasive Group B Streptococcus of Hospital Origin in Malaysia

**DOI:** 10.21315/mjms2020.27.1.14

**Published:** 2020-02-27

**Authors:** Menagah Ezhumalai, AbdulRahman Muthanna, Zarizal Suhaili, Nurul Diana Dzaraly, Syafinaz Amin-Nordin, Mohammad Noor Azmai Amal, Mohd Nasir Mohd Desa

**Affiliations:** 1 Department of Biomedical Sciences, Faculty of Medicine and Health Sciences, Universiti Putra Malaysia, Selangor, Malaysia; 2 School of Animal Sciences, Faculty of Bioresources and Food Industry, Universiti Sultan Zainal Abidin, Terengganu, Malaysia; 3 Department of Medical Microbiology and Parasitology, Faculty of Medicine and Health Sciences, Universiti Putra Malaysia, Selangor, Malaysia; 4 Department of Biology, Faculty of Science, Universiti Putra Malaysia, Selangor, Malaysia

**Keywords:** Streptococcus agalactiae, multilocus sequence typing, human

## Abstract

The aim of this study was to study the genotype of a hospital collection of Group B Streptococcus (GBS) from invasive and non-invasive sites. Fifty-one pre-characterised human of GBS were re-identified and further analysed by multilocus sequence typing (MLST) in relation to previously published serotypes. Fifteen sequence types (ST) were found with ST1 being the most predominant. ST1 was also associated with majority of the invasive isolates. The genotypic distribution patterns of GBS in this study were largely in agreement with previous reports from other countries indicating the tendency of certain genotypes to prevail in human infection settings.

## Introduction

*Streptococcus agalactiae*, also known as Group B Streptococcus (GBS) is a non-motile, Gram-positive cocci in chains or short chains, Christie-Atkins-Munch-Peterson (CAMP) positive, catalase negative and β-haemolytic diplococci bacteria. GBS is one of the most common infectious agents in neonates and pregnant women, and it is a frequent cause of maternal and neonatal sepsis (1, 2). GBS may also cause invasive infections in elderly, nonpregnant populations and immunocompromised patients with underlying medical conditions (3).

GBS has been characterised by 10 capsular serotypes (Ia, Ib, and II to IX) that are distinguished by capsular polysaccharides antigens (CPS) (4). Multilocus sequence typing (MLST) is a genotypic analysis that provides the genetic background information of a bacterial population by sequencing the internal fragments of seven housekeeping genes (1).

To our knowledge, only limited studies have reported on the genotypic distribution of GBS in Malaysia. Therefore, this study aimed to determine the distribution of GBS genotypes and serotypes in a collection of invasive and colonising GBS isolates of hospital origin in Malaysia.

## Methods

### GBS Isolates Collection

This study analysed 51 viable GBS isolates from our previous GBS study (5). These isolates were collected from 2012–2014 at three tertiary hospitals in the Klang Valley, Malaysia. The GBS isolates were categorised into invasive (*n* = 23) and colonising (*n* = 28) isolates based on the isolation sites and the clinical manifestations of the patients.

### Re-identification, Serotyping and MLST of GBS Isolates

The GBS isolates were re-identified using standard phenotypic methods and the following criteria: whitish-grey round colonies with raised edges, a narrow β-haemolytic zone on 5% Columbia sheep blood agar and Gram-positive cocci in chains or short chains. The GBS isolates were also confirmed by a negative catalase test and a positive CAMP test, as indicated by an arrowhead GBS β-haemolytic zone located perpendicular to a *Staphylococcus aureus* ATCC 700699 control.

The capsular serotypes of the isolates had been previously determined using multiplex polimerase chain reaction (PCR) (5). The DNA was extracted using a GeneAll Exgene cell SV mini genomic extraction kit (GeneAll, South Korea). MLST was performed on the 51 GBS isolates, as described by Jones et al. (4). The MLST primer sequences for the seven housekeeping genes were obtained from http://www.mlst.net. The allele number and the sequence type (ST) for each isolate were obtained from the MLST databases (https://pubmlst.org/sagalactiae/).

## Results

### MLST

The sequences of the seven housekeeping genes were successfully amplified in 50 GBS isolates, while that for the remaining one isolate showed only the presence of five out of seven housekeeping genes. The MLST analysis confirmed the presence of allelic profile and ST of 15 different STs. Among the 15 STs, the predominant STs were ST1 (*n* = 21) followed by ST23 (*n* = 7); ST19 (*n* = 4); ST103 (*n* = 3); ST167, ST335, ST28, ST24 (*n* = 2 for each); and ST14, ST17, ST144, ST314, ST651, ST635, ST485 (*n* = 1 for each) (Table 1).

### Distribution of STs among Different Serotypes and Isolation Sites

Many invasive isolates were of ST1 (*n* = 14), followed by ST28, ST23 (*n* = 2 for each); and ST19, ST24, ST144, ST103, ST485 (*n* = 1 for each). Serotype Ia was found to be predominant in ST23 (*n* = 7), followed by ST1 (*n* = 5); ST103 (*n* = 3); ST24 (*n* = 2); and ST144, ST314, ST651, ST485 (*n* = 1 for each). Serotype III was found in ST19 (*n* = 3); ST335 (*n* = 2); ST1, ST167, ST17 (*n* = 1 for each). Serotype IV was found in ST1 (*n* = 6); ST28, ST635 (*n* = 1 for each). Serotype V was found in ST1 (*n* = 6) and ST19 (*n* = 1). Serotype II was found in ST1 (*n* = 2) and ST28 (*n* = 1). Serotype VIII was found in ST1 and ST14 (*n* = 1 for each). One isolate of ST167 was nontypeable (Table 1).

## Discussion

In this study, MLST analyses identified 15 STs among 50 GBS isolates, with ST1 being the most prevalent (42.0% of the strain collection). A study conducted by Jones et al. (4) found that ST1, ST17, ST19 and ST23 made up two-thirds of a strain collection from the United Kingdom, the United States, Japan, New Zealand, Israel, Singapore and Thailand. Tien et al. (6) also reported that ST1 (26.0%) was the most common ST recovered in Taiwan. In contrast, Manning et al. (7) suggested that the most common STs recovered from a set of Canadian isolates were ST23, ST19, ST12, ST17 and ST8 (82.0% of the strain collection).

In this study, the invasive isolates from the three hospital settings were ST1 (60.9%), followed by ST28, ST23 (8.7% for each); ST19, ST24, ST144, ST103, ST485 (4.3% for each). These results are consistent with a study conducted in Taiwan, where the most common invasive STs were ST1 (29.4%), followed by ST12 (20.6%), ST17 (17.6%) and ST23 (14.7%) (6). However, a study from Sweden reported different results, where the invasive STs were ST17, ST19, ST23 and ST1 (8).

In our study, GBS serotypes Ia, II, III, V, VI and VIII were predominant in ST1. These results are consistent with a study by Jones et al. (4), where GBS serotypes Ib, III, V, VI, VIII and the non-typeable serotype were found in ST1. Several studies have reported that ST17 only included serotype III, which was associated with invasive neonatal disease (4, 6, 8–10). Our study found that ST17 included serotype III, but our results showed that this serotype was associated with non-invasive disease in adults. Similarly, a study conducted by Wang et al. (11) showed the predominance of ST17, which comprised 80% of the serotype III invasive GBS isolates. Martins et al. (12) also showed that ST19 comprised a GBS serotype III that was associated with colonising infection. In addition, a study conducted in China reported the prevalence of ST19 among serotype III isolates that caused community- and hospital-acquired infections (13).

Our results were also consistent with previous reports where ST19 colonising isolates comprised serotype III and that ST1 (*n* = 6) and ST19 (*n* = 1) expressed serotype V; these studies also showed that serotype V was primarily distributed in ST1 (6, 10, 14). Since the late 1990s, the emergence of serotype V has been reported among non-pregnant adults in the United States and Sweden (8). Yoon et al. (15) reported that serotype V was the fifth most common serotype, after serotypes Ia, Ib, II and III and that it caused infection in both neonates and adults.

In this study, serotype VI was identified in ST1 (*n* = 6) and ST635 (*n* = 1). A study conducted in Taiwan showed that serotype VI was predominant in CC1 (ST1). Two instances of serotype VIII were reported in this study, correlating to ST1 (*n* = 1) and ST14 (*n* = 1) (16). Jones et al. (4) showed that serotype VIII was also predominant in ST1. Since the 1980s, the prevalence of serotypes VI and VIII has increased among pregnant women in Japan (17, 18). However, a high frequency of serotypes VI and VIII has not been reported in the Western literature. If the incidence of serotypes VI and VIII increases, Lachenauer et al. (17) have suggested that the vaccine formulation should be modified.

In our study, only one serotype could not be typed; this isolate belonged to ST167. Jones et al. (4) also showed that there were non-typeable serotypes in ST1, ST2, ST8, ST10 and ST19. Gherardi et al. (19) found non-typeable serotypes that belonged to ST1, ST10 and ST24. Another study reported that there were non-typeable serotypes in ST28, ST19, ST27 and ST130 (12). Ramaswamy et al. (20) defined a non-typeable serotype as a bacterial isolate that did not react with capsular polysaccharide antisera. This phenomenon could be due to serotype instability, as serotypes may fluctuate over time (6–8). It has also been suggested that horizontal transfer or genetic recombination between serotypes may lead to serotype switching (7).

## Conclusion

The MLST analysis on GBS in this study were largely in agreement with previous reports from other countries with exception of some uncommon STs. This indicates the tendency of certain genotypes to prevail in human infection settings. Further surveillance of GBS from various geographical areas of Malaysia is important for epidemiological studies and vaccine development in Malaysia.

## Limitation

This study has relatively small sample size which limits the evaluation of epidemiological parameters.

## Figures and Tables

**Table 1:** Serotype distribution among the ST and isolation sites

ST	Serotype*							Invasive isolates	Colonising isolates	Total isolates

	Ia	II	III	V	VI	VIII	NT			
19			3	1				1	3	4
1	5	2	1	6	6	1		14	7	21
167			1				1	0	2	2
335			2					0	2	2
28		1			1			2	0	2
14						1		0	1	1
17			1					0	1	1
23	7							2	5	7
24	2							1	1	2
144	1							1	0	1
314	1							0	1	1
103	3							1	2	3
651	1							0	1	1
635					1			0	1	1
485	1							1	0	1
**Total**	**21**	**3**	**8**	**7**	**8**	**2**	**1**	**23**	**27**	**50**

Notes: ST = sequence type; NT = non-typeable serotype

* Suhaimi et al. (5)
